# Optical Coherence Tomography for the Diagnosis of Exercise-Related Acute Cardiovascular Events and Inconclusive Coronary Angiography

**DOI:** 10.1155/2020/8263923

**Published:** 2020-07-22

**Authors:** Caterina Mas-Lladó, Jaume Maristany, Josep Gómez-Lara, Marcos Pascual, María del Mar Alameda, Alfredo Gómez-Jaume, Vicente Peral-Disdier

**Affiliations:** ^1^Department of Cardiology, Health Research Institute of the Balearic Islands (IdISBa), Hospital Universitari Son Espases, Palma, Spain; ^2^Department of Cardiology, Hospital Universitari de Bellvitge, L'Hospitalet del Llobregat, Barcelona, Spain

## Abstract

**Objectives:**

The aim of this study is to assess the utility of optical coherence tomography (OCT) in patients with exercise-related acute coronary syndrome (ACS) presenting with inconclusive angiographic findings.

**Background:**

Regular physical activity reduces the incidence of cardiovascular events. Nevertheless, the risk of ACS or sudden cardiac death (SCD) increases during sport. In adults older than 35 years, exercise-related ACS or SCD is associated with plaque rupture, but not infrequently patients present ambiguous angiographic findings.

**Methods:**

Between September 2015 and January 2020, patients admitted for ACS or SCD triggered by physical exertion and with coronary stenosis ≤50% were included in this prospective observational study. OCT was performed on the artery deemed to be responsible of the event.

**Results:**

Ten patients were enrolled, predominantly men (80%) of middle age (51 years old, IQR 41–63) with low cardiovascular risk burden. Cycling was the most frequent (50%) exercise-related trigger, 8 patients were regular sport practitioners, and 7 had the clinical event during strenuous exertion. Five patients presented with non-ST-elevation ACS, two with ST-elevation ACS, and three with SCD. Angiographic analysis showed nonsignificant stenosis in all patients (42% stenosis, IQR 36–46). OCT identified the etiology of the event in 9 patients (4 plaque erosion, 3 plaque rupture, 1 eruptive calcific nodule, and 1 coronary dissection). Treatment was adjusted according to OCT findings.

**Conclusions:**

OCT is a valuable technique to identify the etiology of exercise-related ACS or SCD in patients with nonobstructive coronary arteries and, as a result, may lead to a more specific treatment.

## 1. Introduction

Regular physical activity and cardiorespiratory fitness both reduce the incidence of cardiovascular events [1–4]. Despite these robust long-term benefits, the risks of acute coronary syndrome (ACS) and sudden cardiac death (SCD) are increased during and shortly after physical exertion, with a relative risk that range from 2.4 to 74 depending on the intensity of exercise and the previous physical condition [5–17]. Indeed, vigorous physical activity increases the risk of acute myocardial infarction and SCD especially among sedentary individuals with previously known or unnoticed cardiovascular disease [5].

Up to 13.6% of acute myocardial infarctions have been related to exertion [5, 12, 13]. In adults >35 years of age, exercise-related ACS and SCD are predominantly associated with underlying coronary artery disease and acute plaque rupture [17–20]. Coronary angiography is often indicated to rule out coronary artery disease in patients admitted for ACS. However, about 5–20% of ACS patients present with nonsignificant stenosis and ambiguous angiographic findings [20–23]. Optical coherence tomography (OCT) is an intravascular image technique with high spatial resolution, permitting to accurately detect unstable plaques, plaque composition, minimal lumen area, and the presence of alternative conditions associated with ACS (i.e., spontaneous coronary dissection, coronary spasm, or myocardial bridging). In addition, OCT has a prominent role in optimizing stent implantation and evaluating causes of stent failure [24–27]. The aim of the present study is to assess the utility of OCT to reach a final etiologic diagnosis in exercise-related ACS and SCD patients with inconclusive angiographic findings.

## 2. Methods

### 2.1. Study Design

This is a prospective, single-center, and observational study. All consecutive patients admitted for ACS or SCD triggered by physical exertion and presenting with nonsignificant angiographic stenosis (≤50% diameter stenosis) were included. All the individuals underwent OCT imaging on the artery deemed to be responsible of the event considering angiographic, electrocardiographic, and echocardiographic data. When necessary, OCT was performed in more than one artery. Percutaneous coronary intervention was left to the operator's criteria after careful revision of the angiographic and OCT images. In patients treated with stents, OCT was repeated to assess the result at the operator's discretion.

Prior physical condition of the individuals and type of sport triggering the ACS were prospectively registered with a questionnaire to assess sport intensity during the event according to the Borg Rating of Perceived Exertion Scale, and previous regular physical activity defined as 150 minutes of moderate aerobic exercise weekly or 75 minutes of vigorous aerobic exercise weekly.

All patients provided written informed consent. The authors declare that all data are available within the article.

### 2.2. OCT and Angiographic Analysis

OCT runs were acquired with the Dragonfly Optis catheter (Abbott, Westford, MA) with automatic pullback of 55 or 75 mm on the target segment. OCT images were analyzed with the OCT Optis Ilumien System (Abbott). Angiographic analysis was performed with Stenosis Analysis 1.6 software (GE Healthcare, Advantage Workstation 4.5, Chicago, IL).

Offline OCT qualitative analysis was performed by two experienced analysts. The following OCT findings were assessed according to previously published expert consensus: [25]

1. Causes of the ACS or SCD: plaque rupture was defined as the presence of an intimal tearing or dissection on the fibrous cap, usually accompanied by the presence of thrombus. Plaque erosion was defined as an exclusion diagnostic in case of white thrombus overlying fibrous plaques without fibrous cap disruption (definite) or in case of irregular luminal surface (with or without thrombus attenuating the underlying plaque) without evidence of superficial lipid or calcification in the vessel upstream or downstream of the thrombus site (probable). Eruptive calcific nodule was recognized when calcific nodules (a signal-poor region with sharply delineated borders) protrude into the intracoronary lumen forming sharp angles and with the presence of a disruption or a thrombus on the surface [28]. Spontaneous coronary dissection was defined as a separation of the intima and media from the adventitia, with or without communication with the vessel lumen [25, 26].
2. Vulnerable plaque findings: thin cap fibroatheroma, presence of macrophages, neovascularization, and cholesterol crystals. Thin cap fibroatheroma was defined as fibrolipidic plaques with an overlying fibrous cap ≤65 *μ*m. Macrophages appeared as signal-rich punctate focal regions that exceeded the background intensity speckle noise. Neovascularization was defined as signal-poor voids within the intima visualized in multiple contiguous frames. Cholesterol crystals were recognized as high-intensity thin linear regions usually associated with a fibrous cap or necrotic core [28].

### 2.3. Statistical Analysis

Statistical analysis was obtained with StataIC 16.0. Quantitative variables are expressed as median and interquartile range (IQR), and qualitative variables are shown in absolute numbers and percentages as frequency measure.

## 3. Results

Between September 2015 and January 2020, 10 consecutive patients who presented with exercise-related ACS or SCD attributed to underlying ACS and stenosis ≤50% on coronary angiography were included. Before coronary angiography, no patient received thrombolytic therapy or thrombus aspiration.

### 3.1. Clinical Characteristics

Table 1 shows baseline characteristics of the study individuals. Median age was 51 years old (IQR 41–63), and 80% of patients were male. Patients had low cardiovascular risk burden: 4 were current or past smokers, 1 had hypertension, 4 had dyslipidaemia, and none was diabetic; in total, 2 patients had 2 risk factors, 5 had one single risk factor, and 3 did not have any. Clinical presentation was ACS without ST segment elevation in 50% of patients, ACS with ST segment elevation in 20%, and SCD in 30%. Nine patients (90%) had a significant high value of ultrasensitive troponin I. Left ventricular ejection fraction was >50% in all. In our series, the most frequent sport triggering acute cardiovascular events was cycling (50% of cases). A majority of the individuals were usual sport practitioners (8 patients). The acute cardiovascular event was related to vigorous exertion in 7 patients, yet 6 of those 7 patients with strenuous exercise as trigger of the clinical event were regular sport practitioners.

### 3.2. Angiographic Characteristics

Median time from initial diagnosis to angiography was 48 hours (IQR 48–72). All patients had at least one nonsignificant stenosis in the vessel deemed to be culprit.  Table 2 summarizes the angiographic data of the study. The most frequently involved coronary artery was the left anterior descending (6 patients). The median coronary stenosis was 42% (IQR 36–46) by quantitative coronary angiography analysis. The angiographic appearance of the stenosis was smooth in 4 patients, calcified in 1, mildly irregular in 4, and irregular with possible thrombus in 1. Therefore, angiography suggested an unstable plaque in this last patient only.

### 3.3. OCT Findings

OCT was conducted in a total of 11 arteries. Median minimal luminal area was 3.0 mm (IQR 2.6–4.7). A total of 8 patients had atherosclerotic plaques responsible of the clinical event: plaque erosion was documented in 4, plaque rupture in 3, and eruptive calcific nodule with thrombus and intimal discontinuity in 1 patient. Thrombus was detected in 7 patients. OCT showed two or more characteristics of vulnerable plaque in 50% of patients. One patient had spontaneous coronary dissection, and only one patient did not have any of the aforementioned OCT findings.  Table 3 shows the OCT findings.

### 3.4. Treatment Decision

The 8 patients with atherosclerotic culprit plaques were treated with percutaneous coronary intervention and drug-eluting stent implantation. The other two patients were medically managed. OCT was repeated for stent assessment in 6 of the 8 patients treated with stents: 1 case of stent underexpansion was treated with balloon postdilation. Stent malapposition, uncovered struts, or stent edge dissection was not detected. In-hospital evolution was excellent in all patients, without any major adverse cardiovascular event.

**Figure 1:**
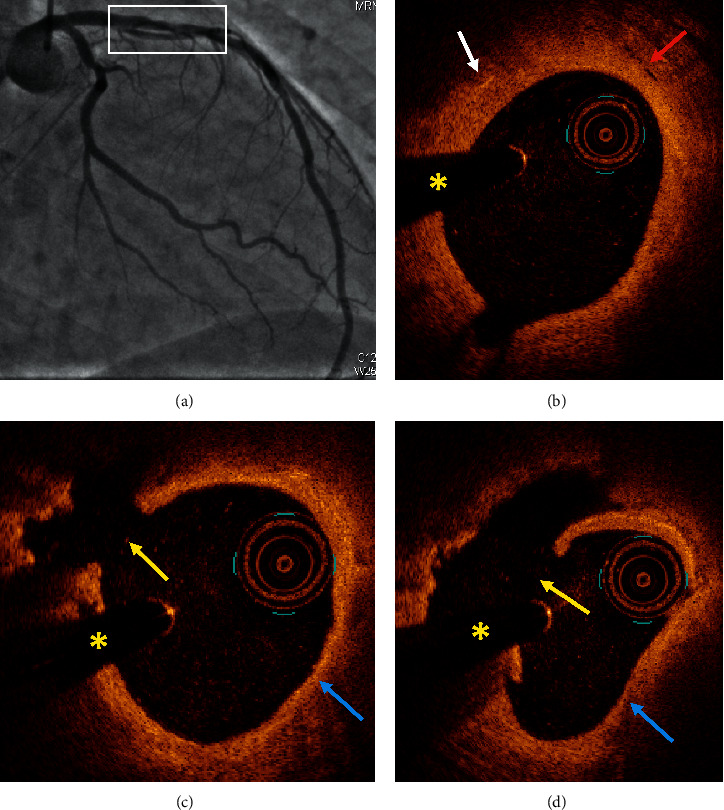
Case #2. Plaque rupture. (a) Coronary angiography demonstrated a nonsignificant stenosis in the mid-left anterior descending artery (white box). (b) OCT analysis showed a fibrolipidic plaque with possible cholesterol crystals (white arrow) and neovascularization (red arrow). (c, d) OCT revealed a plaque rupture (yellow arrow) and a thin cap fibroatheroma (blue arrow). ^*∗*^Shadow caused by the wire. OCT: optical coherence tomography.

**Figure 2:**
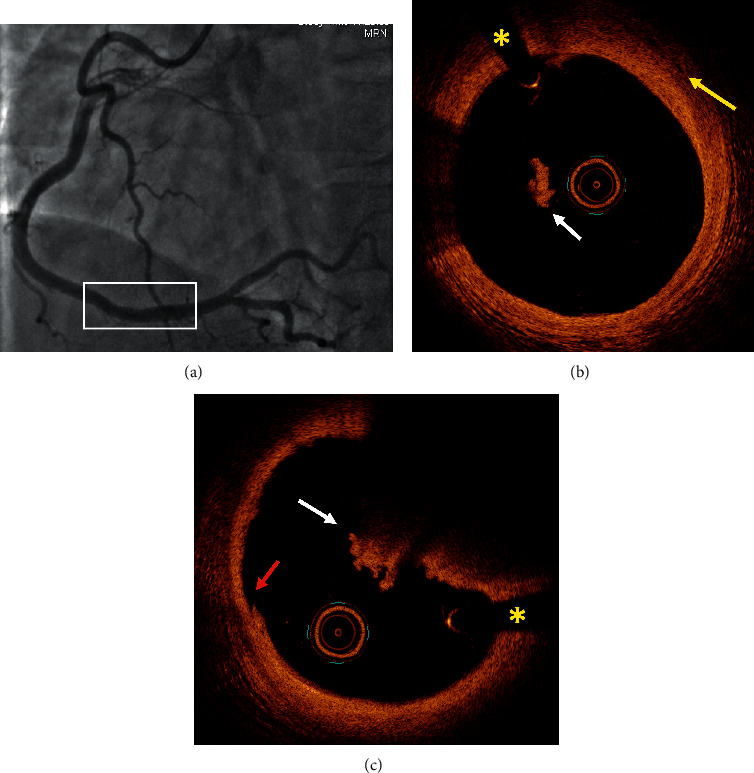
Case #4. Plaque erosion. (a) Coronary angiography showed a mildly irregular nonsignificant stenosis in the distal right coronary artery. (b) OCT demonstrated a fibrous plaque with neovascularization (yellow arrow) and intravascular thrombus (white arrow). (c) OCT revealed a large thrombus (white arrow) with irregular luminal surface (red arrow) in continuity with the fibrous plaque. Intimal tear was not detected. ^*∗*^Shadow caused by the wire. OCT: optical coherence tomography.

**Figure 3:**
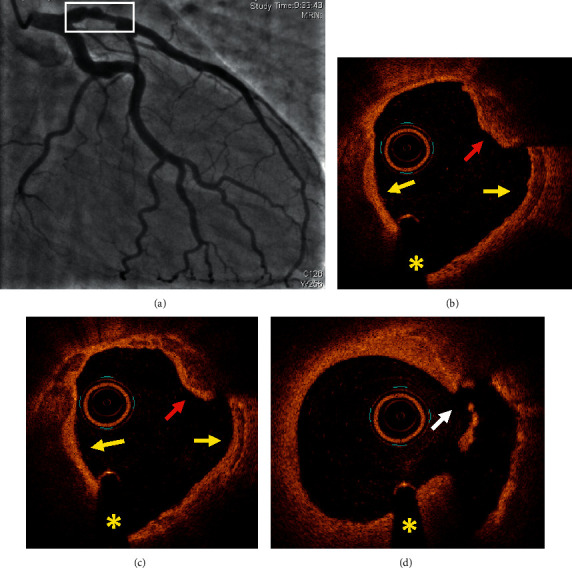
Case #6. Eruptive calcific nodule with plaque rupture. (a) Coronary angiography demonstrated an irregular plaque with nonsignificant borderline stenosis in the proximal left anterior descending artery. (b, c, d) OCT findings: fibrocalcific plaques (yellow arrows), one eruptive calcific nodule protruding into the lumen (red arrow) associated with plaque rupture (white arrow). ^*∗*^Shadow caused by the wire. OCT: optical coherence tomography.

**Figure 4:**
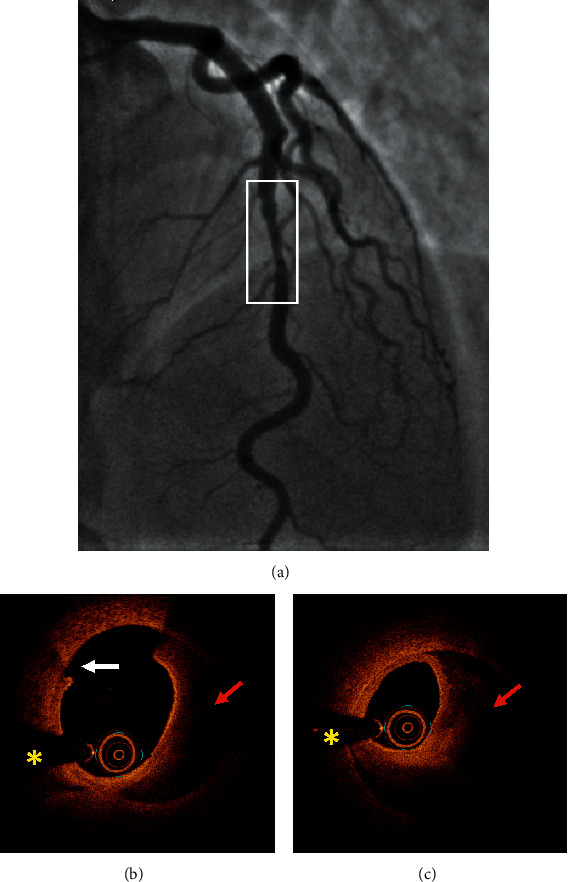
Case #10. Spontaneous coronary artery dissection. (a) Coronary angiography showed an irregular nonsignificant stenosis in the mid-left anterior descending artery. (b, c) OCT revealed an intimal tear (white arrow) and intramural hematoma (red arrow) surrounding the intimomedial membrane. ^*∗*^Shadow caused by the wire. OCT: optical coherence tomography.

Table 4 summarizes all the data of the 10 patients. Figures 1–4 show representative angiographic and OCT images of 4 patients enrolled in the study.

## 4. Discussion

Some previous investigations described clinical and angiographic characteristics of exercise-related ACS and SCD [5, 19, 20, 22, 29], but to our knowledge, this is the first OCT study in this particular subgroup of sport-triggered ACS and SCD patients with nonsignificant stenosis on angiography.

The individuals of our study were predominantly middle-aged men with low cardiovascular risk burden in agreement with prior studies [5, 15, 19, 20, 30]. Remarkably, 80% of the included patients were usual sport practitioners, particularly endurance sports such as cycling. In contrast, Mittelman et al. found that sedentary subjects were more prone to exercise-related ACS compared to regular sport practitioners [5]. Previous works connected long-term endurance exercise with increased coronary calcification, although it is not clear whether it poses an increase in cardiovascular risk or not [31–33]. However, in our series of patients, OCT showed very scarce calcified plaques. Iannaccone et al. observed that thin cap fibroatheroma was a strong predictor of plaque rupture in patients with ACS (it was detected in 77% of ACS with ST segment elevation and in 60% of ACS without ST segment elevation) [34]. In the present study, thin cap fibroatheroma was detected in 4 patients (40%): one ACS with ST segment elevation, two ACS without ST segment elevation, and one SCD. In our series, patients with thin cap fibroatheroma presented different mechanisms of plaque disruption: 1 case with plaque rupture, 2 with plaque erosion, and 1 with eruptive calcific nodule. Unfortunately, there are no OCT studies in stable sport practitioners to further elucidate the atherosclerotic plaque burden of this population.

Left anterior descending artery was the most frequently involved artery, as previously published in the exercise-induced ACS setting [35]. One aspect to take into consideration is that all the analyzed patients had nonsignificant stenosis on angiography both by visual and quantitative coronary angiography. Minimal luminal area assessed by OCT confirmed nonsignificant area reductions in all the individuals as well [36]. Moreover, angiography only suggested unstable plaque in one patient. Due to the high spatial resolution for characterization of atherosclerotic plaques and detection of culprit lesions [24–26, 28, 37–39], OCT imaging permitted a final etiologic diagnosis in 9 of the ten patients: atherosclerotic culprit plaques in 8 and coronary dissection in 1 individual. The value of OCT to identify plaque disruption and vulnerable plaques has already been published in myocardial infarction with nonobstructive coronary arteries (MINOCA) patients, but not in an exercise-induced ACS scenario [40]. In addition, a recently published OCT study described plaque characteristics in exercise-induced non-ST segment elevation ACS in patients with clearly significant angiographic stenosis [41]. Differently, the scope of our study was to assess the usefulness of OCT to obtain further diagnostic information in patients with inconclusive angiographic findings.

The operators decided to implant stents in all the patients with atherosclerotic culprit plaques. Nevertheless, whether stent implantation or medical approach is more appropriate to treat unstable nonsignificant plaques is a controversial issue [42–44]. In this cohort of sport-triggered acute cardiovascular events, we consider of paramount value the role of OCT to identify the underlying etiology to prescribe a more tailored therapy and avoid unnecessary or unspecific treatments, such as implantable defibrillators for false “primary” SCD, aggressive antithrombotic regimens or stent implantation in spontaneous coronary dissections, or patients without plaque disruption. Previous studies have already shown that an OCT-guided approach may improve outcomes in patients with acute coronary syndromes [45]. Furthermore, OCT is not only a technique for plaque characterization or to guide treatment but for stent assessment too [25, 27, 37].

Finally, our study has some limitations. First, due to the acute setting of patients with exercise-induced ACS or SCD, it is possible that some critically ill patients or presenting in nonworking hours may have been not enrolled in the present study. Second, the profile of our study individuals is probably selected, as there was a strong suspicion of acute coronary event of atherosclerotic origin according to clinical presentation, electrocardiography, echocardiography data, and in whom angiography showed some degree of nonsignificant stenosis to guide the location of OCT pullbacks. This could explain the excellent performance of OCT to identify the etiology of the ACS in 9 of the 10 patients. And last, the overall study follow-up was short because six of the ten patients were lost from follow-up after hospital discharge as they were tourists from a foreign country. The evolution of the remaining four individuals was excellent (median follow-up of 14 months, IQR 5–22, with no adverse cardiovascular events).

## 5. Conclusion

Optical coherence tomography is a valuable technique to identify the etiology of exercise-related ACS or SCD in selected patients with nonobstructive coronary arteries and thus may lead to a more specific therapy.

## Figures and Tables

**Table 1:** Baseline characteristics (*n* = 10).

Age in years, median (IQR)	51 (41–63)
Male sex, *n* (%)	8 (80)
Smoker, *n* (%)	4 (40)
Hypertension, *n* (%)	1 (10)
Dyslipidaemia, *n* (%)	4 (40)
Diabetes mellitus, *n* (%)	0 (0)
Diagnosis on admission, *n* (%)	
ACS without persistent ST segment elevation	5 (50)
ACS with ST segment elevation	2 (20)
SCD	3 (30)
Ultrasensitive troponin I, *n* (%)	
No elevation	1 (10)
<1000 ng/L	4 (40)
>1000 ng/L	5 (50)
LVEF, median (IQR)	65.5 (63–70)
Type of physical exertion, *n* (%)	
Running	2 (20)
Cycling	5 (50)
Hiking	1 (10)
Isometric exercise	2 (20)
Sport intensity related to the acute cardiovascular event, *n* (%)	
Moderate	3 (30)
Vigorous	7 (70)
Usual sport practitioners, *n* (%)	8 (80)
Months of follow-up, median (IQR)	1 (1–10)
Death, recurrent AMI, or revascularization on follow-up, *n* (%)	0 (0)

ACS: acute coronary syndrome; AMI: acute myocardial infarction; IQR: interquartile range; LVEF: left ventricle ejection fraction; SCD: sudden cardiac death.

**Table 2:** Angiographic findings.

Time in hours from initial diagnosis to angiography, median (IQR)	48 (48–72)

Suspected culprit coronary artery, *n* (%)	
LAD	6 (60)
RCA	3 (30)
CX	0 (0)
Unknown	1 (10)
Visual coronary stenosis in (%), median (IQR)	40 (30–40)
QCA stenosis in (%), median (IQR)	42 (36–46)

Angiographic lesion characteristics, *n* (%)	
Smooth	4 (40)
Calcified lesion	1 (10)
Irregular	4 (40)
Irregular with thrombus	1 (10)

CX: circumflex coronary artery; IQR: interquartile range; LAD: left anterior descending coronary artery; QCA: quantitative coronary angiography analysis; RCA: right coronary artery.

**Table 3:** OCT findings.

Minimal luminal area in mm^2^, median (IQR)	3.0 (2.6–4.7)

Cause of the ACS, *n* (%)	
Unknown	1 (10)
Plaque erosion	4 (40)
Plaque rupture	3 (30)
Eruptive calcific nodule with plaque disruption	1 (10)
Coronary artery dissection	1 (10)
Thrombus, *n* (%)	7 (70)
Stable plaque, *n* (%)	1 (10)

Vulnerable plaque findings, *n* (%)	
Thin cap fibroatheroma	4 (40)
Macrophages	4 (40)
Neovascularization	6 (60)
Cholesterol crystals	2 (20)

ACS: acute coronary syndrome; IQR: interquartile range; OCT: optical coherence tomography; SCD: sudden cardiac death.

**Table 4:** Individual characteristics of the study population.

	Clinical characteristics								Angiographic characteristics			OCT characteristics		
Case	Sex	Age	RF	Physical exertion	REG	DX	Pre-TX	usTrI (ng/L)	CCA	QCA (%)	Lesion characteristics	Detected cause	Vulnerable plaque	TX decision
1	M	63	HBP	Cycling	No	SCD	None	716	RCA	43.8	Calcified	Rupture	NV	PCI
2	M	46	SMK and DLP	Isometric	Yes	NSTEMI	Aspirin, ticagrelor, and enoxaparin	446	LAD	36.2	Irregular	Rupture	NV, MC, CC, and TCFA	PCI
3	F	59	None	Running	Yes	SCD	Aspirin, ticagrelor, and enoxaparin	43	UK	45.7^*∗*^	Smooth	None	None	PHARM
4	M	40	SMK and DLP	Cycling	Yes	STEMI	Aspirin and ticagrelor	5000	RCA	31.5	Irregular	Erosion	NV	PCI
5	M	56	None	Cycling	Yes	SCD	Aspirin, clopidogrel, and enoxaparin	1700	LAD	36.4	Smooth	Erosion	NV, MC, and TCFA	PCI
6	M	69	SMK	Cycling	Yes	NSTEMI	Aspirin, ticagrelor, and enoxaparin	293	LAD	40.2	Irregular	Eruptive calcific nodule	MC, CC, and TCFA	PCI
7	M	81	DLP	Hiking	Yes	NSTEMI	Aspirin, clopidogrel, and enoxaparin	1450	RCA	43.2	Smooth	Erosion	NV and MC	PCI
8	M	41	SMK	Isometric	No	NSTEMI	Aspirin, ticagrelor, and fondaparinux	3070	LAD	49.7	Smooth	Rupture	None	PCI
9	M	33	DLP	Cycling	Yes	STEMI	Aspirin and clopidogrel	29264	LAD	37.0	Thrombus	Erosion	NV and TCFA	PCI
10	F	41	None	Running	Yes	NSTEMI	Aspirin and ticagrelor	149	LAD	46.0	Irregular	Dissection	None	PHARM

CCA: culprit coronary artery; CC: cholesterol crystals; DLP: dyslipidaemia; DX: diagnose on admission; F: female; HBP: high blood pressure; usTrI: ultrasensitive troponin I; LAD: left anterior descending coronary artery; M: male; MC: macrophages; NSTEMI: non-ST-elevation myocardial infarction; NV: neovascularization; OCT: optical coherence tomography; PCI: percutaneous coronary intervention; PHARM: pharmacological treatment; pre-Tx: in-hospital treatment prior to coronary angiography; QCA: quantitative coronary angiography; RCA: right coronary artery; REG: regular sport practitioner; RF: risk factors; SCD: sudden cardiac death; SMK: smoker; STEMI: ST-elevation myocardial infarction; TCFA: thin cap fibroatheroma; TX: treatment. ^*∗*^QCA of the maximal stenosis detected on that patient, despite there was no suspicion of which plaque was the culprit lesion.

## Data Availability

The data used to support the findings of this study are included within the article.
